# Editorial: Tumor microenvironment and personalized therapy of gastrointestinal cancer

**DOI:** 10.3389/fmed.2025.1560642

**Published:** 2025-02-21

**Authors:** Xinxin Wang, Jianming James Tang, Shuang Yang, Shuo Li

**Affiliations:** ^1^General Surgery Department, Chinese PLA General Hospital, Beijing, China; ^2^School of Medicine, Nankai University, Tianjin, China; ^3^Department of Medicine/Division of Infectious Diseases, University of Alabama at Birmingham (UAB) Schools of Medicine, Birmingham, AL, United States

**Keywords:** personalized therapy, gastrointestinal cancer, immunotherapy, tumor microenvironment, targeted molecular therapy

In recent years, the tumor microenvironment in gastrointestinal cancer has emerged as a focal point of research due to its indispensable role in tumor initiation, progression, and metastasis. This intricate microenvironment, composed of diverse cellular and non-cellular components, undergoes continuous and dynamic interactions that not only reshape tumor immunity but also significantly influence the effectiveness of therapies, including immunotherapy and molecular targeted therapy. Despite the growing application of these therapeutic approaches, evidenced by increased response rates and improved survival outcomes in broader patient populations, critical challenges persist, such as unsatisfactory efficacy, the lack of precise prediction of effectiveness, and drug resistance. A comprehensive understanding of the tumor microenvironment is key to uncovering the mechanisms of tumor progression, facilitating personalized therapeutic strategies, and driving the innovation of novel treatment strategies.

The present Research Topic focused on the clinical practice of immunotherapy and molecular targeted therapy in gastrointestinal cancer. It aimed to dissect the tumor microenvironment and establish robust methods for predicting efficacy, providing a theoretical foundation to enhance the prognosis and quality of life for patients with gastrointestinal cancer.

Gambella et al. conducted a comprehensive evaluation of YKL-40 levels in a multicenter cohort of 72 patients diagnosed with anal squamous cell carcinoma. They utilized immunohistochemistry staining to assess YKL-40 expression in both tumor cells and peritumor immune cells within surgical resection specimens. Additionally, they analyzed the serological levels of YKL-40 at the time of diagnosis and during follow-up visits. The findings indicated promising prospects for YKL-40 as both a prognostic marker based on serum levels and a predictive marker associated with tissue protein expression in peritumor immune cells for anal squamous cell carcinoma.

Liu et al. performed a multi-omics analysis incorporating bulk RNA sequencing, single-cell RNA sequencing, and whole-exome sequencing to explore the tumor microenvironment of left-sided (LCC) and right-sided colon cancer (RCC). Results revealed significant differences in immune cell infiltration between LCC and RCC. Whole-exome sequencing identified a notably higher mutation frequency in RCC compared to LCC. Single-cell analysis highlighted RCC-specific tumor cell subclusters, alongside CD8+ T cell populations in RCC showing exhaustion and recent activation, indicative of tumor-specific cytotoxic T lymphocytes (CTLs). Furthermore, intercellular communication analysis showed more frequent and intense interactions between tumor-specific CTLs and tumor cells in RCC.

Cui et al. undertook a study involving 177 patients undergoing neoadjuvant chemotherapy and immunotherapy followed by gastrectomy. Uni- and multivariable logistic regression models identified risk factors for postoperative complications, and a predictive nomogram was developed. Significant risk factors included age ≥70 years, higher blood loss, platelet/lymphocyte ratio ≤ 196, neutrophil/lymphocyte ratio >1.33, non-R0 resection, and body mass index < 18.5 kg/m^2^. The nomogram, based on these factors, demonstrated strong predictive performance and good calibration.

Pelosi et al. analyzed the metabolome composition of the secretome in a three-dimensional co-culture model of colorectal cancer (CRC) and adipocytes. This approach, integrating advanced biological techniques, marked a significant step forward in understanding CRC-adipocyte interactions. The study identified key lipids (PG 20:0, 11Z-Octadecenal, 9,10-Dihydroxy-12Z-octadecenoic acid, Palmitoleic acid, PA 18:4) and an amino acid derivative (Acetylglutamic acid), providing insights into potential biomarkers and the underlying mechanisms of CRC-adipocyte crosstalk.

Tong et al. studied clinicopathological features and follow-up data of a retrospective cohort of 1,757 cases of stage 0–IV colorectal cancer and revealed significant differences in certain clinicopathological features. The sCEA, tCEA, and combined CEA demonstrated prognostic value in stages III-IV of CRC, with only combined CEA acting as an independent prognostic factor in these stages. Therefore, combined CEA might be considered a novel indicator for evaluating CRC prognosis.

